# Wave Speed in Excitable Random Networks with Spatially Constrained Connections

**DOI:** 10.1371/journal.pone.0020536

**Published:** 2011-06-03

**Authors:** Nikita Vladimirov, Roger D. Traub, Yuhai Tu

**Affiliations:** IBM T. J. Watson Research Center, Yorktown Heights, New York, United States of America; University of Maribor, Slovenia

## Abstract

Very fast oscillations (VFO) in neocortex are widely observed before epileptic seizures, and there is growing evidence that they are caused by networks of pyramidal neurons connected by gap junctions between their axons. We are motivated by the spatio-temporal waves of activity recorded using electrocorticography (ECoG), and study the speed of activity propagation through a network of neurons axonally coupled by gap junctions. We simulate wave propagation by excitable cellular automata (CA) on random (Erdös-Rényi) networks of special type, with spatially constrained connections. From the cellular automaton model, we derive a mean field theory to predict wave propagation. The governing equation resolved by the Fisher-Kolmogorov PDE fails to describe wave speed. A new (hyperbolic) PDE is suggested, which provides adequate wave speed 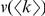 that saturates with network degree 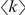, in agreement with intuitive expectations and CA simulations. We further show that the *maximum* length of connection is a much better predictor of the wave speed than the *mean* length. When tested in networks with various degree distributions, wave speeds are found to strongly depend on the ratio of network moments 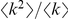 rather than on mean degree 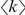, which is explained by general network theory. The wave speeds are strikingly similar in a diverse set of networks, including regular, Poisson, exponential and power law distributions, supporting our theory for various network topologies. Our results suggest practical predictions for networks of electrically coupled neurons, and our mean field method can be readily applied for a wide class of similar problems, such as spread of epidemics through spatial networks.

## Introduction

Different types of networks are found across many scales, from metabolic networks in a single cell, to neural networks in brain, up to social and technological global networks. The theory of networks receives increasing attention since the pioneering works that formulated random graphs [1], and the recently discovered ubiquity of small-world networks [2] and scale-free networks [3]. Reviews on general theory of networks can be found in [4]–[6]. A comprehensive up-to-date review of spatial networks is given in [7].

Since its first formulation [1], the Erdös-Rényi (ER) graph became a cornerstone of network theory. An ER graph 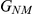 consists of 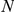 nodes and 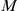 links (edges), and each link connects two nodes which are selected randomly. In a sufficiently large network, the number of links emanating from a node (*degree*) is a random variable with Poisson distribution 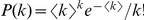, where 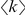 is the network mean degree, 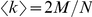. Despite its advantages, the ER graph is not suitable for studying *spatial* phenomena because it is spatially homogeneous. However, in most real-world networks the connections are spatial and variable in length. Also, the maximum length of connection is usually limited by the available resources or other natural restrictions. To address this problem, spatial generalizations of the ER graph were suggested. For example, two nodes can be connected only if the distance between them is below threshold 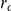 [8]. This model was used to simulate spatio-temporal activity in networks of electrically coupled neurons [9]. Another example is the Waxman model, in which the probability that two nodes are connected is a decreasing function of distance between the nodes [10]. The latter model was used to simulate the Internet [11].

In many networks the nodes are excitable, meaning that active state can arise and propagate from one node to another if they are connected. In this way, action potentials propagate through neural networks, computer viruses spread in the Internet, and diseases are transmitted through transport networks. If the nodes are excitable, dynamical states propagate through a network both temporally and spatially, leading to waves and more complex patterns.

**Figure 1:**
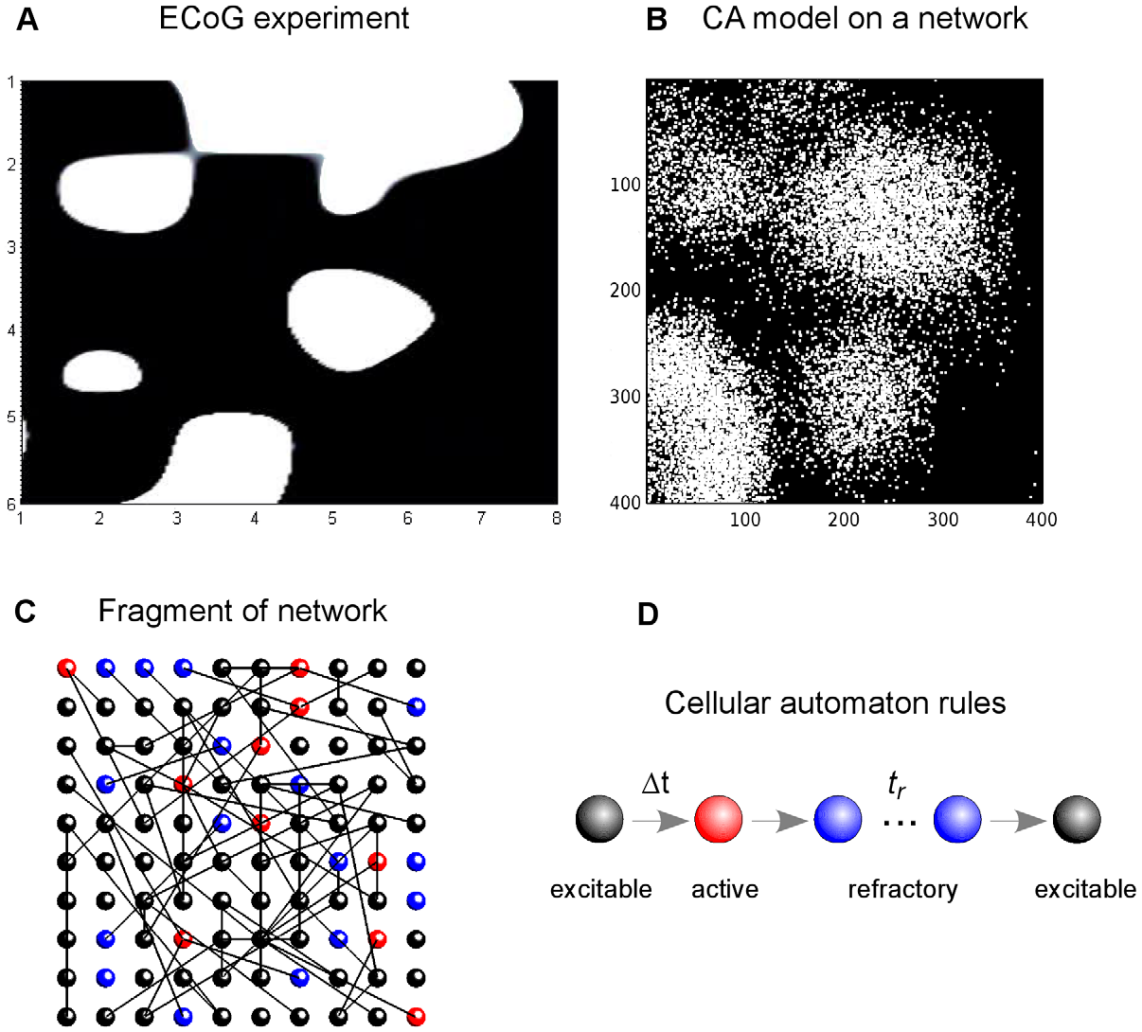
Neural network activity in experiments and in the cellular automaton model. A. A snapshot of electrocorticographic (ECoG) data of brain activity, measured by 8×6 subdural array of electrodes. Data is interpolated between nodes, white areas correspond to high activity. B. A snapshot of activity from a cellular automaton model in an 400×400 network. The network is subject to noisy input from spontaneously activating cells (rate 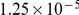). Active cells are white, refractory and excitable are black (simplified color code). C. Snapshot of activity in a 10×10 sub-network with detailed color code: red for active, blue for refractory, black for excitable nodes. Lines show links between nodes. D. Rules of the CA model: excitable node (black) may become active (red), if activated by a neighbor. After being activated, the node becomes refractory (blue) for a period of time 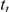, after which it becomes excitable again. Data in A is a courtesy of Miles Whittington, recorded in Patient B of [29].

A case study in our work is the emergence of spatiotemporal patterns with very fast oscillations (VFO, 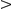
80 Hz) measured by electrocorticography [9], recorded in neocortex of patients prior to epileptic seizures (Figure 1A). There is growing experimental and theoretical evidence that VFO are caused by electrically coupled pyramidal neurons which are connected by gap junctions, thus providing direct excitation from one to another, which does not require synaptic transmission [9], [12], [13].

Nodes in such a network are dynamical excitable units. Although their intrinsic behavior can be complex and require detailed multi-compartment model of each neuron [14]–[16], understanding of the neural network spatiotemporal oscillations requires highly reduced models, such as cellular automata which capture three main states (resting, firing, refractory) with a minimal set of parameters [13]. The system can be described by a *network* version of Greenberg-Hastings cellular automaton (GHCA), a discrete model of an excitable medium [17]. Variations of GHCA have been used in many studies, including collective oscillations of pyramidal cells in the hippocampus [8], [13], [18], sensory networks [19], [20], and the evolution of HIV infection [21].

In the network version of GHCA that we use, cellular interactions can be distant rather than next-neighbor [8]. The cells are connected into Erdös-Rényi random graph with spatially constrained connections (hereafter SCC), so the distance between connected nodes is not greater than connectivity radius 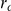. Under random spontaneous activation of some of the resting cells, large networks demonstrate oscillatory dynamics with complex spatio-temporal activity driven by many interacting waves. Traub and coauthors [9] have recently demonstrated these patterns to be strikingly similar to those observed in ECoG recordings (Figure 1B). In the model, the complex patterns of activity arise when multiple waves are born from spontaneously activated cells and they grow and coalesce in time and space.

A single active node may generate an expanding circular wave of excitation, if the mean network connectivity is high enough. The wave maintains its shape and travels with constant speed, which is an important characteristic of system's excitability and depends on network topology. Knowledge of wave speed in excitable networks allows prediction of how fast the active state (neuronal activity, viral infection) propagates and invades the rest of the network. Although simulations can be done in each particular case, it is important to have a basic mean field theory (MFT) to understand and predict the network dynamics without simulations.

In this paper we derive an MFT to predict wave speed in a random (Erdös-Rényi type) network with spatially constrained connections (SCC), for given connectivity radius 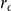 and mean degree 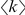. The results are generalized to Erdös-Rényi type networks with various radii distributions, and further to non-Erdös-Rényi networks with various degree distributions, suggesting universality of our mean field theory.

## Results

### The system

In the following section we describe the system and its dynamic properties, along with simulations that demonstrate its typical behavior.

#### 2D network

We study a 2D network of excitable cells (nodes), which are are set on a uniform 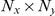 grid, with unit space between adjacent nodes (Figure 1C). Connections (links) are bidirectional, i.e. activity can be transmitted in both directions. Links can be long but limited by 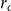: within a circle of radius 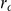 (network with round ‘footprint’) or within a square with side 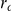 (network with square ‘footprint’) [8]. Generally, the connectivity radius 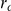 is much larger than 1, but much smaller than array dimensions 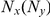. The number of links per node (*degree*) follows the Poisson distribution 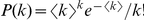, whereas the distribution of link lengths is uniform in (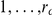), unless stated otherwise.

#### Quasi-1D network

To treat the system analytically, we reduce it to a quasi one-dimensional network: the length of links is limited only along X but may be unlimited in the Y coordinate. In other words, it is a 2D network which has an interval footprint (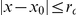). A quasi-1D network provides the same wave speed along X as the 2D network with square footprint (with same 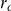), which is also consistent with 2D network with round footprint (simulations not shown).

#### Cellular automaton model

A node in excitable state 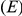 becomes firing 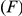 if one or more of its connected neighbors in the network are firing. After one time step 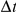 the firing node becomes refractory 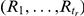 for a relatively long period 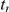, after which it becomes excitable again. Thus each node rests in 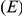 or undergoes a sequence of states 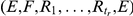 if activated by a neighbor (Figure 1D). Initially all nodes are in excitable state, except a small number of firing nodes that initiate the wave. Node states are updated simultaneously at each time step.

**Figure 2:**
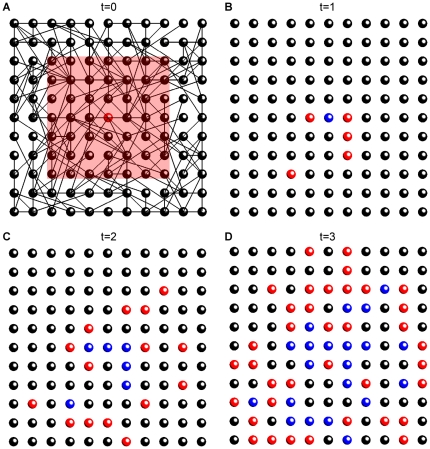
Initiation of wave in a CA model on a random network. The first 4 time steps of wave initiation are shown for an 11×11 network. A. t = 0; B. t = 1; C. t = 2; D. t = 3. Colorcode: red for active, blue for refractory, black for excitable cells. Lines show links between cells, red square shows the connectivity footprint of the central cell (shown only in A). Parameters: 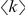 = 4, 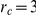 (small for demonstration purposes).

**Figure 3:**
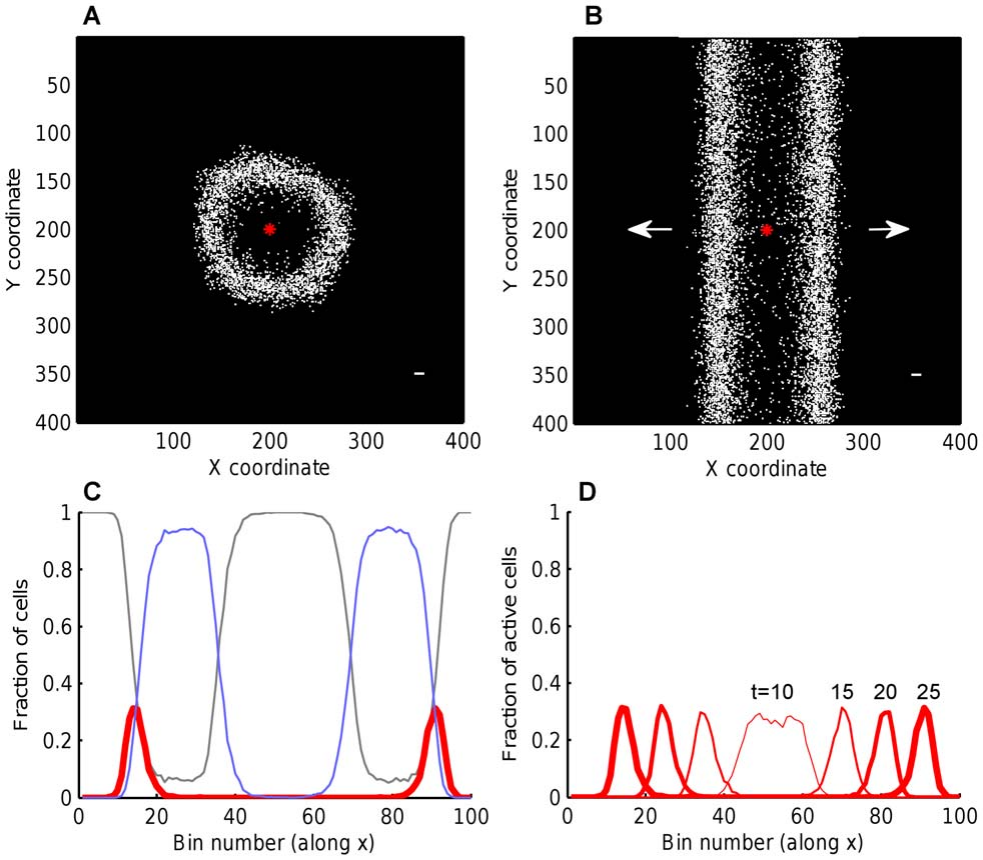
Traveling waves of activity in random networks. Traveling waves emerging in the CA model on random networks with A. square, B. quasi-1D connectivity footprint. The cell which initiates the wave is shown by a red asterisk. Active cells are white, refractory and excitable cells are black. Directions of wave propagation are shown by arrows. C. A snapshot of wave (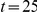) with spatial profiles of all three states: grey for excitable, bold red for active, light blue for refractory cell density. In the center, the wake of excitable cells (grey) grows by recovering from the refractory state (blue). D. Profiles of the active state at four time steps, showing two traveling waves emerged from a single active cell. Once formed, the speed and width of a wave remain constant. Profiles were calculated by averaging active cell counts over 100 bins along X. Parameters 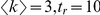, 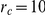 (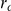 is shown in the bottom right corners in A,B).

Initiation of wave in a small 2D network is shown in Figure 2 (first four time steps). Although directions of links are random, activity propagates outwards from the initial point because it is followed by refractory state, prohibiting backward propagation. Propagation of waves in large 2D networks is shown in Figure 3A–C. Waves in networks with round and square footprint do not differ qualitatively (not shown), because neighbors of each node are chosen as random lattice points from round or square neighborhood (respectively), which differ only in relatively small corner areas.

In the rest of the paper, we work with quasi-1D networks, because they allow transparent mean field analysis, and yet behave almost identically to 2D networks, with the only difference that wave fronts are linear rather than circular (Figure 3B vs. A). Next, we start from simpler case where all links have maximum length 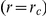, and then analyse the case where links are distributed randomly 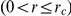.

### Mean field theory

#### Links of maximum length

In order to treat the system analytically, we assume that the network is regular (all nodes have same degree 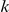), and all links have same length (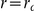). Later we will show that these crude assumptions provide a good approximation for more general cases.

We assume that each excitable cell can become firing by one (and only one) of its neighbors that is firing, which is true for wave front where firing cells are rare. Each firing cell is surrounded by excitable cells, and may produce at maximum 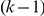 firing neighbors. One of 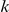 links is missing because it points to a cell from which firing has come. In other words, if a cell is excitable (probability 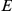), it can be activated by one of its neighbors (probability 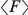, spatial average of 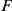). Otherwise, if a cell is already firing (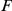) and there are excitable neighbors (prob. 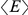), it produces 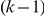 firing cells. These relations can be written as
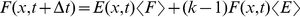
(1)where 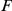 and 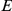 are the probabilities of a cell to be in firing or excitable state, respectively, 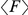 and 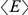 are spatially averaged 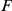 and 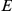 in the neighborhood of 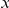. This equation holds only for wave front, where firing cells are scarce, excitable cells are abundant, and there are no refractory cells yet.

With the notion that most cells are excitable 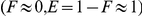, the E-terms become unitary (linearized equation). In the quasi-1D system, 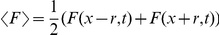, and the equation simplifies to
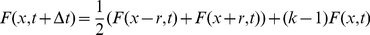
(2)Taylor's expansion in 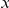 at the right hand side gives
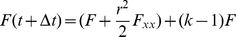
(3)Taylor's expansion in 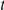 at the left hand side gives
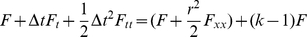
(4)


#### Parabolic (Fisher-Kolmogorov) equation

**Figure 4:**
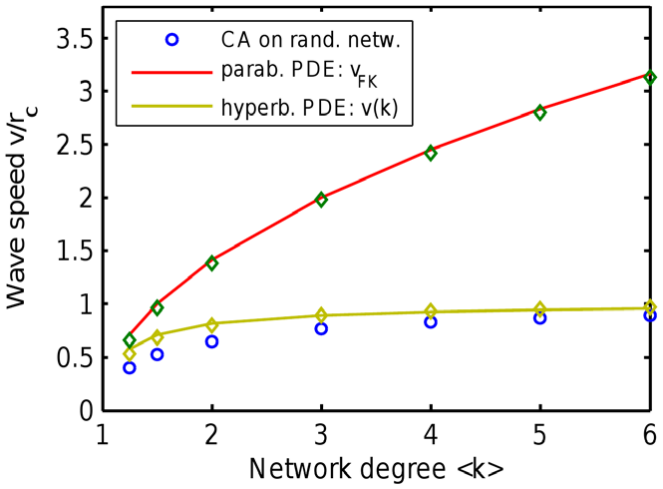
Wave speed predicted by the parabolic and hyperbolic PDEs compared to simulations of CA on random networks. The parabolic (Fisher-Kolmogorov) PDE gives wave speed 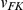 that indefinitely grows with network degree (red line and diamonds). In contrast, the suggested hyperbolic PDE (given in text) provides a reaso`nable wave speed 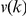 (given in text, shown by green line and diamonds). The 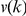 grows moderately and saturates to the maximum possible speed 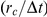, in agreement with CA simulations (blue circles) and intuitive expectations. The solid lines show analytic formulae, the diamonds show simulations of corresponding full PDE systems.

Taking into account only the first time derivative, we arrive at a linearized version of Fisher-Kolmogorov equation
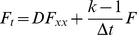
(5)where 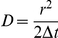 is a diffusion coefficient [22], 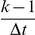 is the growth rate, and the second-order extinction term is omitted for wave speed analysis. The well-known [23] formula for Fisher-Kolmogorov wave speed 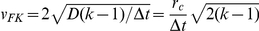 gives infinite growth of speed with mean degree 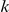 (Figure 4, upper line). Taking high-order terms into account in the right hand side of the PDE does not alter the principal behavior of wave speed (simulations not shown), demonstrating that parabolic PDEs are not suitable for wave speed prediction.

#### Hyperbolic equation

Keeping both first and second time derivatives in the left part of Eqn. 4, we obtain a hyperbolic equation for firing node density
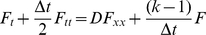
(6)Wave speed can be found by marginal stability analysis [24]. Substituting variable 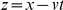, we obtain the equation 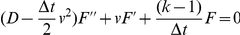. Solution in the form 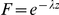 yields a characteristic equation 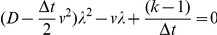. The roots of the characteristic equation must be real, which gives the minimum wave speed
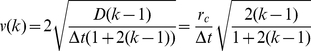
(7)We put the time step 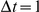 as in the CA model, and 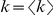. This wave speed demonstrates qualitative agreement with the CA model on an ER SCC network (Figure 4, lower line). Most importantly, 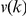 gradually saturates to the maximum possible speed 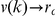 for high 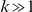, in agreement with CA simulations and intuitive expectations.

**Figure 5:**
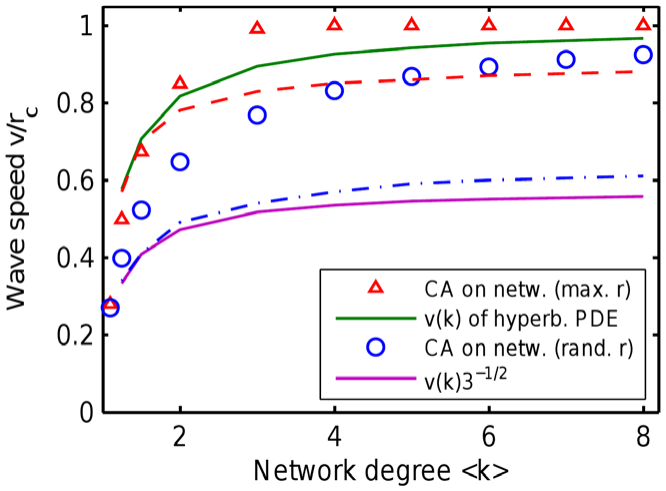
Wave speed derived from the hyperbolic PDE compared to CA simulations. The wave speed 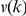 (red line, high) is derived assuming all links have maximum length. CA simulations are shown in two variants, with maximum-length links (red triangles) and generic random-length links (blue circles). The naive speed scaling 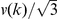 (blue line, low) is derived assuming that link lengths are uniformly distributed. This discrepancy is explained in Results, showing that maximum-length link is a better predictor of wave speed. The dashed lines show the high-order analysis, proving that the hyperbolic PDEs capture the wave behavior sufficiently well, and derivatives of order above 2 are not necessary.

The wave speed 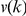 is shown in more detail in Figure 5 (upper solid line). One can see that 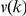 falls near to CA simulations on networks where all links have maximum length 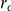 (Figure 5, triangles), as expected. Surprisingly, 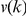 also approximates well the CA simulations on networks where links have random length 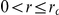 (Figure 5, circles), which is our primary model. This phenomenon is explained in the next section.

### MFT for links with random length

The case where links have random length 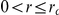 is derived similarly to the maximum-length case shown above. Recall that the spatial scale between nodes is unity, 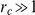 and 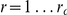. Let all lengths be equally probable, 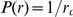. All nodes have degree 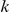, so the fraction of nodes with at least one link of length 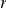 is 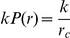. The evolution of firing nodes density in time is given by
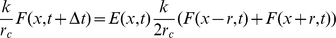


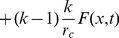
(8)Integration over 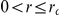 and omitting 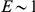 gives
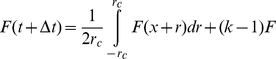
(9)Taylor's expansion up to second derivative of the function under the integral gives the equation
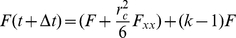
(10)


The latter equation is equivalent to Eqn. 3, with the only difference being a reduced radius (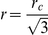). Therefore, the wave speed for the random-length links would be simply scaled by 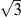 relatively to 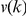: 
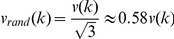
.

**Figure 6:**
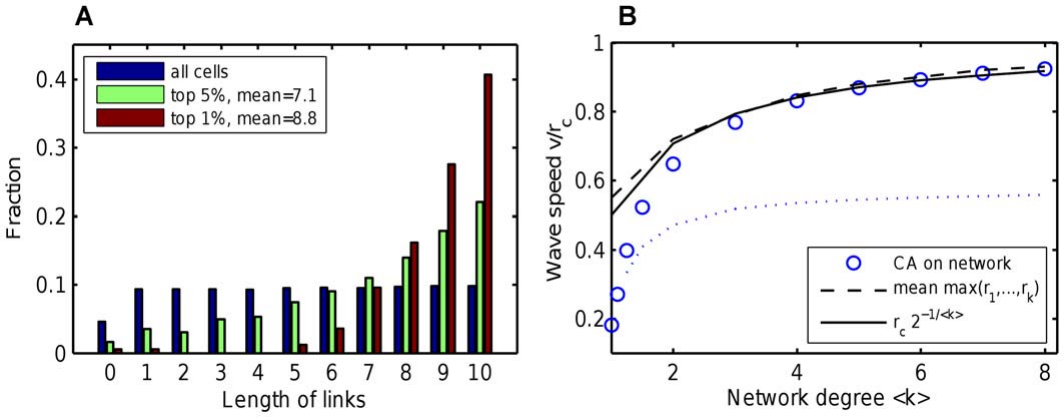
Role of maximum link lengths in wave propagation. A. The distribution of link lengths between the cells at the wave front, and the cells which triggered their firing. The front cells (top 1 or 5 %) were selected by their positions in a wave. The mean distances are given in the legend, parameters 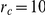, 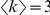. B. Estimate of the wave speed by numerical estimate of mean maxima of 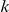 i.i.d radii taken from uniform distribution 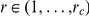 (broken line). The formula 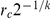 is the expected value of the mean maxima (solid line). The CA simulations of wave speed are shown by circles. As seen, the mean maxima give a good wave speed estimate, in contrast to naive scaling 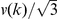 derived earlier from the PDE (dotted line).

However, this naive scaling based on uniform radii distribution underestimates the wave speed of CA roughly by a factor of two (Figure 5, lower solid line, compare to circles). This happens because cells in the wave *front* have actually non-uniform distribution of links from which they have received their activation (Figure 6A). The distribution is strongly biased towards the longest links. A wave front generates a new wave front at the next time step by sending activity through the longest links out of available 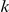. To support this notion, we generated sets of 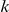 i.i.d. discrete random variables 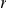 uniformly distributed in 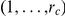 and computed their mean maxima 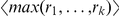 as a plausible estimate of wave speed, that is a contribution of activity propagation from each single node to a global propagation of wave per unit time. As one can see in Figure 6B (broken line), the mean maxima of 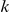 random radii gives a good measure of CA wave speed (circles), especially for high 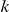. These numerical calculations are supported by analytic formula for the expected mean of maxima (black solid line), 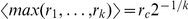, derived for a uniform continuous distribution 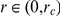. This formula gives a very good prediction of CA wave speed at high 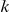, demonstrating that the wave propagation is indeed mainly determined by the mean maximum of 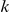 radii, which converges in the limit (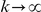) to the maximum possible radius 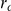. In other words, it is not the average, but rather the *maximum* link length that determines the wave speed in a random network with random radii distribution.

#### Role of link length distribution

**Figure 7:**
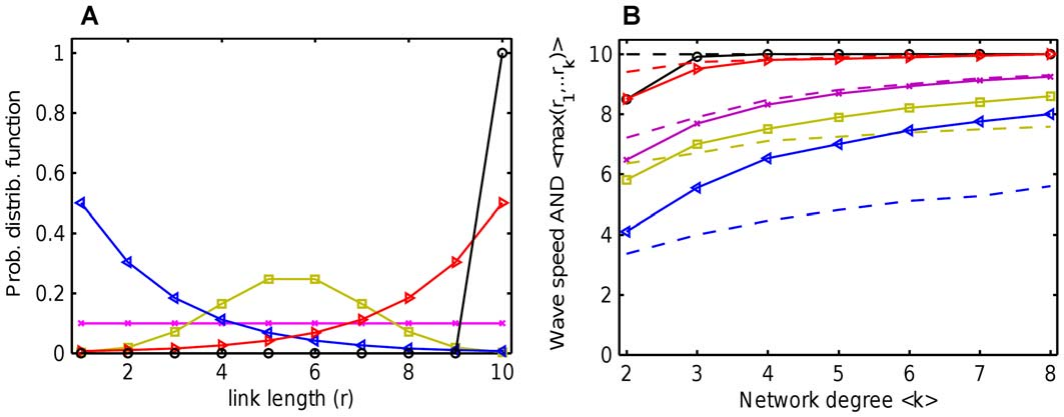
Effect of link lengths (radii) distribution on wave speed. A. The radii distributions between nodes in the random networks: black o - fixed value, cyan x - uniform, red (blue) triangles - exponentially increasing (decreasing), green squares - bell-shaped distribution (see Methods for detailed formulae). B. Wave speeds in the networks with corresponding radii distributions (markers are consistent with panel A). Broken lines are computed mean maxima out of 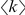 radii samples from each distribution, used as a plausible estimates of the true wave speeds (solid lines). Networks are Erdös-Rényi SCC, so the degree distribution (links per node) is Poissonian.

To study the effects of other possible radii distributions, we simulated networks with five distributions (Figure 7A): the uniform radii distribution, the fixed-value distribution (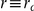), a bell-shaped and two exponential distributions (increasing and decreasing, respectively). The speeds of wave propagation in resulting random networks are qualitatively similar and always significantly higher than the average value of the corresponding distribution (Figure 7B). Broken lines in Figure 7B are mean maxima of 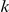 radii generated from each distribution, which served as a good estimate of wave speed in four out of five distributions. Note that the distribution for which our maximum link hypothesis works the least is the one with an exponentially small probability of reaching the maximum link length 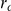 (see the purple line in Figure 7A).

Although we can not provide an analytic estimate of the effective radius in each distribution, these simulations support the notion that wave speed is predominantly determined by the maximum rather than average radius, with maximum taken out of 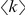 radius realizations.

### High-order analysis

The use of Taylor's approximation in the PDE derivation could potentially bring unwanted errors because 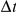 and 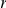 are in fact not small. We use a high-order analysis to estimate wave speed in exact terms, by looking for a traveling wave solution in the form 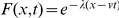 without using Taylor's expansion. See Methods and Algorithms for details. Figure 5 shows that wave speed 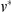 (upper dashed line) obtained by this method is close to 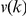 obtained from a hyperbolic PDE (upper solid line). This confirms that considering derivatives of order above 2 (in time and space) will not affect the wave speed qualitatively. Therefore, the hyperbolic PDE is both necessary and sufficient for qualitative wave speed prediction.

### Role of degree distribution

The variation of node degrees depends on degree distribution of a network and strongly affects the wave speed. To ultimately simplify the network and add the variance in stages, we simulated the CA model on spatial networks the following degree distributions:

- regular network (each node has same degree 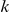),

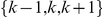
 distribution: a node's degree is chosen randomly from 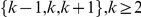,
- Poisson distribution (ER graph), 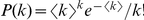

- exponential distribution 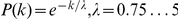,
- power-law distribution with exponential cutoff 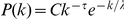 and parameters:

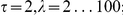

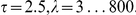

**Figure 8:**
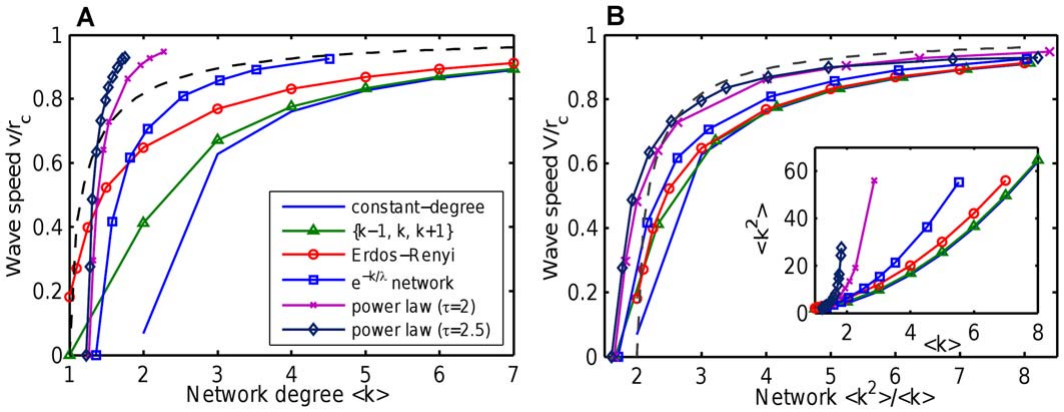
Wave speeds in networks with different degree distributions. Wave speeds in networks of six different degree distributions (explained in legend) are plotted A. against mean degree 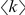; B. against ratio of network moments 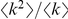. Note the convergence of wave speeds in B. The mean field formulae are shown in both panels by broken lines (Eqn. 7 in A; Eqn. 11 in B). *Inset.* The 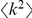 versus 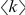 in the simulated networks. Line markers are consistent with legend in panel A. Errorbars are smaller than symbols, due to simulations on many networks of large size (1000×1000).

In all networks, the lengths of links between nodes were uniformly distributed in (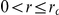). As one can see in Figure 8A, the wave speed profiles vary widely when plotted against network mean degree 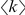. However, they merge into nearly the same shape when plotted against 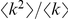 (Figure 8B, inset). The key role of 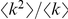 ratio is evident from general network theory. For a randomly chosen link, the degree of a node on its end follows the nearest-neighbor distribution 
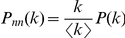
, where 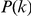 is the original network degree distribution [6]. The mean degree of a *connected* node 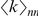 is therefore different from a randomly picked node: 
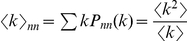
, so our ratio is merely the nearest-neighbor mean degree. In other words, when activation travels from one node to another, the degree of a node in the end of a link follows the nearest-neighbor distribution 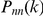, which has a mean of 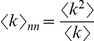 and gives the actual branching ratio of the activation in the new node.

For an ER graph, 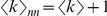. Substitution of 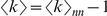 into wave speed (7) gives us a more general formula for fitting wave speeds
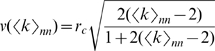
(11)where 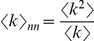 as before. As shown in (Figure 8, inset), this formula gives a good estimate of wave speeds in a variety of network degree distributions.

## Discussion

In summary, we have analysed behavior of excitable random networks with spatially constrained connections (SCC), and derived a mean field theory of the activity propagation. We conclude that the hyperbolic PDE
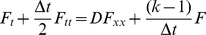
(12)is necessary and sufficient to capture the wave speed in random (uncorrelated) networks with spatially constrained connections.

The wave speed is mainly determined by the longest possible connection between the firing node and a node it can activate, so the mean maximum of 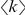 random radii 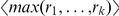 serves as a good predictor of wave speed in Erdös-Rényi SCC networks with various radii distributions.

We have derived formula (7) for wave speed 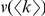, which agrees with simulated behavior of CA on Erdös-Rényi SCC networks. Simulations of CA on networks with other (non-Poisson) degree distributions suggest a more general formula (11) for wave speed, which depends on 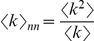, the nearest-neighbor mean degree. So, our mean field theory extends to networks with various degree distributions, provided that 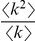 is used as a more universal measure of network's average branching, rather than simple 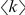, which is explained by general network theory.

The original [8], [9], [13] cellular automaton model of very fast brain oscillations assumed that pyramidal neurons are connected via gap junctions into an Erdös-Rényi SCC network. Our results show that wave speed (hence network excitability) will scale with radius 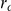 and degree 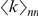 the same way even if the network topology is different. This has experimental implications: accounting for the wave speed does not require reconstruction of full gap junction network topology. Rather, determining the mean 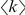 and variance 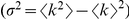 of the gap junctions per cell, and the maximum inter-neuronal distance 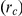 for coupled cells should suffice. Also, our simulations show that wave speed is primarily driven by the longest of 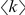 connections emanating from an average node. Therefore knowledge of the longest axoaxonal connections is more important than recovering their full range.

Our present knowledge of wave speed will facilitate a detailed analysis of waves interaction and coalescence, which generate complex oscillatory dynamics in large networks shown in [9].

In general perspective, the fact that wave speed depends on 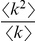 shows that variation in node degree plays an important role in wave propagation. High variation of node degrees provides high wave speed, presumably due to presence of highly-connected nodes (hubs).

The hyperbolic PDE (12) should not be confused with similar-looking telegrapher's equation, because the 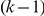 term in Eqn 12 is positive and gives a self-sustaining wave in time, in contrast to the decaying solution of telegrapher's equation.

The special type of networks we study (with spatially constrained connections) should not be confused with small-world networks. The limited length of link is crucial for spatial phenomena, whereas in conventional small-world networks the “short-cuts” are unlimited in length. This makes wave speed infinite and destroys spatial coherence, but improves temporal coherence [25], [26]. However, small-world networks might be constructed with spatially constrained shortcuts, which could be an interesting system for analysis.

The CA model we use is similar to epidemiological SIRS model (susceptible-infected-recovery-susceptible). Therefore, our theory may help predict spatial spread of epidemics in large networks. For example, it may be applied to predict spatial spread of SIR type malware through a sufficiently large network of WiFi routers [27], where the length of connections is limited by the router's range.

We studied networks with degree distributions that cover a broad spectrum of possible 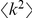 versus 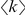 combinations (Figure 8B, inset), suggesting that our MFT holds for uncorrelated networks of arbitrary degree distribution (with spatially constrained connections). The degree distributions studied here appear in real-world networks, such as the neuronal network of *C. elegans*, the power grid network, acquaintance networks and the WWW (see [4] and references therein). Our MFT apply to these and other networks, provided there is a metric to measure connection length, and connections are limited by some constant 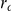.

## Methods

### Cellular automaton model

A node in excitable state 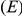 becomes firing 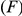 if one or more of its neighbours are 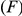. After one time step 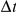 the firing 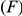 node becomes refractory 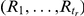 for a relatively long period 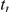, after which it becomes excitable 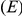 again. Thus each node rests in 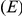 or undergoes a sequence of states 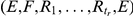 if activated by a neighbor. The formal rules of CA are as follows

1. excitable (E) 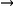 firing (F) if any neighbor is (F)
2. F 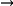 refractory (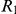)

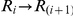

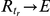

The states of all nodes are updated simultaneously every time step. Initially all nodes are in excitable 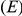 state, except a small number of firing nodes that initiate the wave.

### Network with spatially constrained connections

The network consists of excitable nodes, which are are set on a uniform 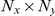 grid, with unit space between adjacent nodes. The network with defined degree distribution 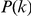 and connectivity radius 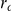 is constructed by a procedure similar to that for spatially homogeneous networks [28]. Initially, each node is assigned to a random number of ‘stubs’ 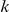, which is picked from distribution 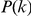. Next, the program picks nodes from a randomized (shuffled) list of all nodes. The list of nodes must be randomized to avoid artificial correlations imposed by node order. For each picked node with nonzero number of stubs, the program randomly picks one of its neighbor within distance 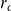. If the neighbor has nonzero number of stubs, too, both nodes are linked. Their numbers of stubs are decremented by -1, and their numbers of links are incremented by +1. The procedure is repeated until all stubs of the chosen node become links. To avoid infinitely long search in a situation when all neighbors are already linked and they have no more free stubs, the search of potential neighbors is stopped after 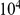 unsuccessful attempts. The procedure is repeated for each node.

The 2D network differs from the quasi-1D network only by the way that the connectivity distance is measured. In 2D network, connectivity of node 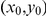 is 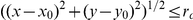 (round ‘footprint’) or 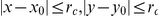 (square ‘footprint’). In quasi one-dimensional network, 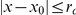 (interval footprint). In most simulations 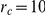, if not specified otherwise.

Single networks of size up to 400
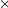
400 were simulated in Matlab. Large-scale simulations of multiple (64 to 128) networks of size 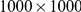 with 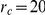 (used in Figure 8) were carried out in C/C++ MPI program on an IBM Blue Gene supercomputer.

### High-order analysis

The use of Taylor's approximation in space and time could potentially bring unwanted errors because 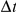 and 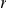 are in fact not small. Here we estimate wave speed in exact terms by looking for a traveling wave solution in the form 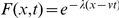 without using Taylor's expansion, thus taking all high-order terms into account. Both cases of links with maximum and random length are analyzed below.

#### Maximum length

Substitution of 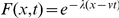 into Eqn. 2 gives
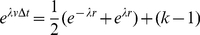
(13)For convenience, we will change to new variables 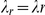 and 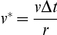. The equation then reads 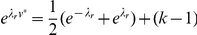 and can be solved numerically for marginal stability analysis.

In order to find the minimal wave speed, we need to find 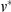 such that the function in the left part 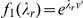 has a unique common point with the function in the right part 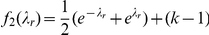, for given 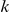. This happens when 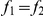 and 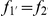 (plots touch each other). Parameter 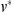 is the minimal wave speed, normalized to the radius 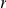. Figure 5 shows that 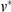 (upper dashed line) obtained by this method is close to 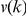 obtained from a hyperbolic PDE (upper solid line), so considering derivatives of order above 2 (in time and space) will not affect the wave speed qualitatively. Therefore, the hyperbolic PDE is both necessary and sufficient for qualitative wave speed prediction.

#### Random length

This case is analyzed in a similar way. Eqn. 2 linearized around the unstable steady state reads: 
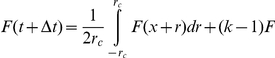
. Substitution of 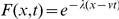 and changing to 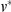 and 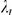 gives
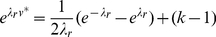
(14)This equation is solved numerically in the same way as in the maximum-length case. As one can see in Figure 5, the obtained minimal wave speed 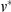 (lower dashed line) is close to 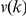 obtained in hyperbolic PDE (lower solid line). However, as shown in Results, the MFT for maximum link length provides better approximation of CA simulations, so this case is shown here only for completeness of analysis.

### Radii distributions

We studied five different radii distributions: uniform in 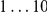, fixed-value (always 10), bell-shaped 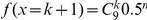 with binomial coefficients 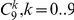, and two exponentially shaped distributions 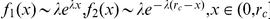, both normalized to compensate the cutoff of exponential tails.

### Numerical integration of PDE system

Analytic formulas for wave speeds are supported by numerical integration of corresponding PDE systems. In the hyperbolic case, the closed nonlinear system reads
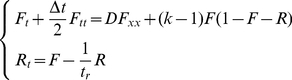
(15)By the normalization 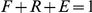 the equation for 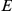 is not necessary. Parameters are 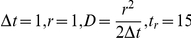. This system was replaced by a system of three parabolic PDEs (by introducing 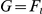), which was solved by a method of lines in Matlab, with parameters 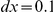, 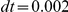, mesh size 200
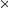
200, and explicit Euler scheme. Initial conditions were 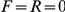 in all points except the center line where 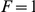.

Simulations of traveling waves in the Fisher-Kolmogorov equation were carried out similarly, with the first equation changed to 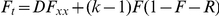.

Both systems demonstrate the effect of wave coalescence – two waves cancel each other at the areas where they meet, while their outer borders merge into one big circular wave. The main difference between the two PDE systems is the speed of wave, which grows infinitely with 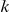 in the Fisher-Kolmogorov system, but saturates to unity for the hyperbolic PDE system, as discussed in the Results.
